# Muscle-brain crosstalk as a driver of brain health in aging

**DOI:** 10.1007/s11357-025-01833-0

**Published:** 2025-08-15

**Authors:** B. L. McNeish, I. Miljkovic, T. Liu-Ambrose, F. Ambrosio, K. Esser, M. Fahnestock, C. Rosano

**Affiliations:** 1 Department of Physical Medicine and Rehabilitation, University of Michigan, Ann Arbor, MI USA; 2 Department of Physical Medicine and Rehabilitation, University of Pittsburgh, Pittsburgh, PA USA; 3 Department of Epidemiology, School of Public Health, University of Pittsburgh, Pittsburgh, PA USA; 4 Department of Physical Therapy, University of British Columbia, Vancouver, BC Canada; 5 Department of Physical Medicine and Rehabilitation, Harvard Medical School, Boston, MA USA; 6 Department of Physiology and Aging, University of Florida, Gainesville, FL USA; 7 Department of Psychiatry and Behavioural Neurosciences, McMaster University, Hamilton, ON Canada

**Keywords:** Myosteatosis, Brain health, Alzheimer’s disease, Myokines, Extracellular vesicles, Muscle-brain crosstalk

## Abstract

Cognitive impairment and dementia in older adults represent significant global health challenges. Although the bidirectional relationship between physical function and brain health is well established, the mechanistic drivers of this link remain poorly understood. Muscle function and quality are central to physical function, and muscle’s secretome is increasingly recognized for its systemic health effects—supporting the potential for muscle-to-brain crosstalk. This concept was explored at the 3rd International Research Symposium on Brain Health, jointly hosted by Vancouver Coastal Health and the University of British Columbia. We present the findings of this symposium, which reviewed the current state of the literature on muscle-to-brain crosstalk from multiple perspectives, spanning population studies to preclinical models. A key focus was the muscle secretome, particularly myokines and extracellular vesicles, as potential messengers influencing brain health. The symposium also identified critical takeaways and proposed next steps to further elucidate the underlying mechanisms of muscle-to-brain crosstalk and explore how these pathways might be harnessed through exercise or pharmacologic interventions to promote brain health in older adults.

**Supplementary Information:**

The online version contains supplementary material available at 10.1007/s11357-025-01833-0.

## Introduction

Late-life cognitive impairment and dementia impose soaring costs on the individual, the community, AND the public health system [1]. The complexity of dementia etiology taxes our traditional models of inquiry, limiting our ability to develop interventions that effectively prevent it or slow down its disease course. A novel and integrative paradigm is needed to identify modifiable targets for interventions for late-life cognitive impairment and dementia.

**Fig. 1:**
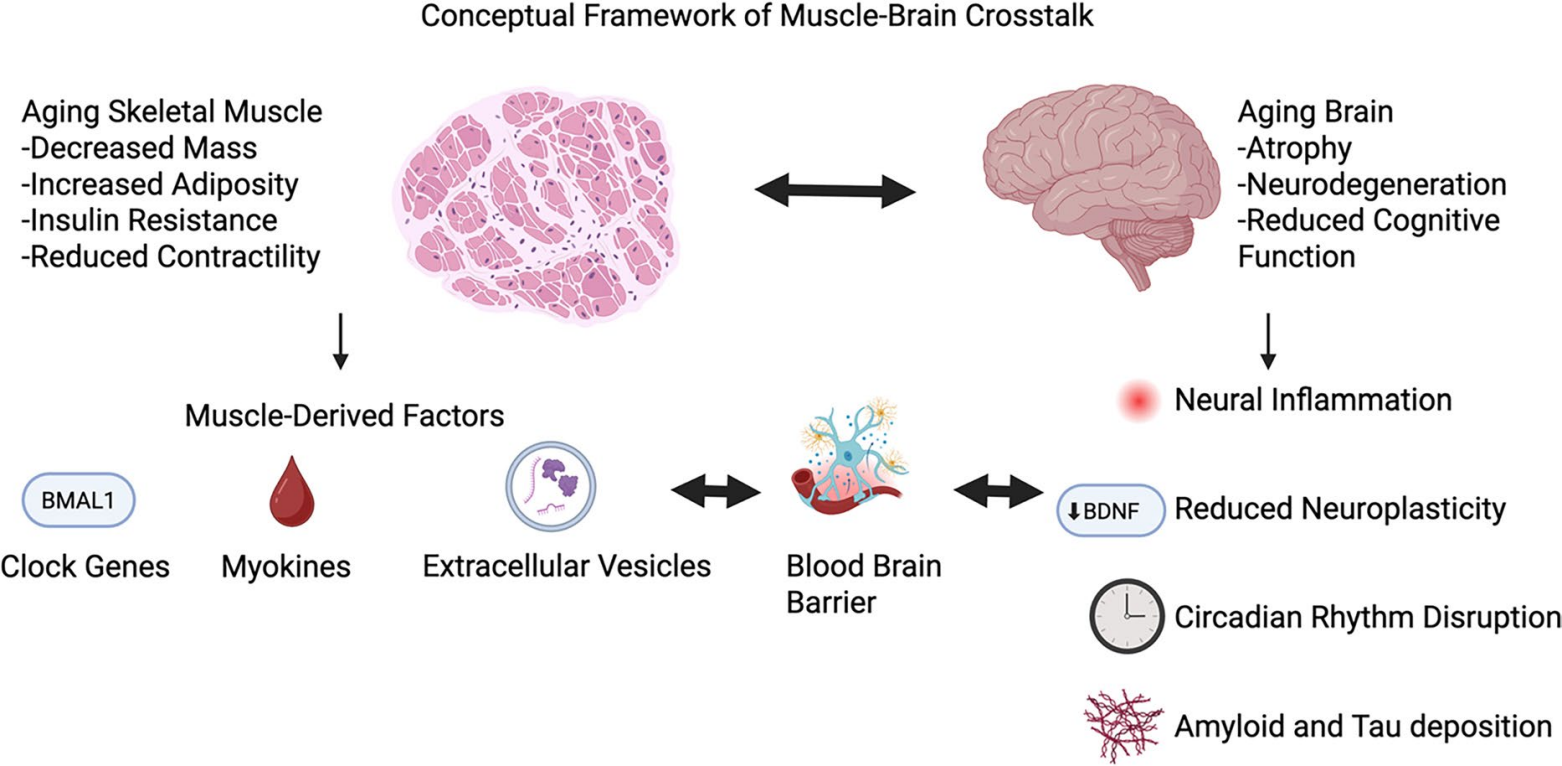
Conceptual framework of muscle–brain crosstalk. Aging skeletal muscle secretes muscle derived factors—myokines, extracellular vesicles, and clock gene products—that influence neural inflammation, neuroplasticity, circadian rhythms, and amyloid/tau pathophysiology. Bidirectional communication underlies the bidirectional association between muscle and brain health outcomes

There is a well-established bidirectional relationship between physical activity, physical function, and brain health in older adults. Skeletal muscle, a key driver of physical activity and function, is now emerging as a novel source of neuroprotection in aging; it is also a promising target for preventing and treating cognitive impairment and dementia. Observational studies show muscle function and quality are associated with cognitive function and neuroimaging indices of brain integrity [2, 3]. Specifically, it is now recognized that skeletal muscles produce and secrete circulating factors including nucleic acids, fatty acids, peptides, and proteins known as myokines. Myokines have been the subject of major inquiry and have been shown to possess neuroprotective effects [4, 5]. Myokines include cytokines or peptides synthesized by myocytes in muscle tissue [6]. Myokines mediate communication between muscle and other organs, including the brain (i.e., muscle-brain cross-talk), as well as within the muscle. For example, accumulating evidence suggests that the myokine cathepsin B passes through the blood–brain barrier to enhance local brain-derived neurotrophic factor production, enhancing neurogenesis, memory, and learning [7, 8]. It has been suggested that myokines may, in part, underlie the benefits of physical activity on the central nervous system. More recently, circulating extracellular vesicles (EVs), which comprise a plasma membrane that encapsulates molecular cargoes, are emerging as carriers of signaling molecules between muscle and brain. Emerging research on the relationship between sleep and cognitive health suggests that the circadian clock within skeletal muscle plays a significant role in maintaining overall circadian integrity and regulating sleep—both of which have important implications for brain health. Figure 1 presents a conceptual framework for muscle–brain crosstalk, integrating key mechanisms, signaling molecules, and bidirectional communication across systems.

To date, the majority of studies on muscle-brain crosstalk have been performed among individuals at the extremes of muscle health spectrum, such as healthy individuals who are able to engage in high-intensity or volume of exercise, or individuals with neuromuscular disease (i.e., Duchenne’s Muscular Dystrophy) [9]. Despite impaired mobility and cognitive impairment being recognized as “geriatric giants,” there is a dearth of studies focused on community-dwelling older adults.

To harness the potential of muscle-brain crosstalk in maintaining or promoting brain health in aging, the Centre for Aging SMART at Vancouver Coastal Health and the University of British Columbia in Vancouver held the 3rd International Research Symposium on Brain Health: *The Role of Muscle-Brain Crosstalk to Promote Healthy Aging*. The symposium, funded by the Canadian Institutes of Health Research, offered a critical review of inter-related disciplines in aging, with a focus on cognitive and muscle health, physical activity/exercise, neuroscience, and functional genomics. Topics encompassed current knowledge of the molecular links between muscle and brain aging and a review of mechanisms that are critical for muscle health and interventions that stimulate muscle contractility. The findings and recommendations of this symposium are presented here. Our objective is to summarize the muscle-brain crosstalk evidence relevant to brain aging and to provide recommendations for future research directions.

In the “Myosteatosis: a marker of muscle quality, aging and disease” section, we review current knowledge on muscle’s physiological changes with age and the neuroprotective effects of physical activity in older adults. In the “Epidemiological association of muscular contractile activity and brain health” section, we review evidence that both aerobic and resistance exercise benefit brain health, but highlight a key gap in understanding the biological mechanisms—particularly how different exercise types alter the muscle secretome. In the “Mechanisms of muscle-brain crosstalk: circadian rhythms, muscle clocks, myokines and extracellular vesicles” section, we examine how circulating mediators—such as myokines and EVs—influence brain health from systems-level (e.g., circadian rhythms) to molecular-level neurotrophic effects. We review recent work on EVs in aging models, pointing to novel messaging between muscle and brain and the therapeutic potential of EV engineering. Lastly, in the “Discussion/conclusions” section, we synthesize insights from the first three sections, highlight crosscutting themes, and outline future research directions to advance understanding of muscle–brain crosstalk and its therapeutic application. Supplementary Table 1 lists the presenters for the four sections from the symposium *Muscle–Brain Crosstalk as a Driver of Brain Health in Aging*.

**Table:** 

Critical knowledge gaps the symposium aimed to address:
How do changes in muscle quality—specifically myosteatosis—influence brain health?
Which characteristics of muscle might underlie the known cognitive benefits of physical activity?
Given muscle’s emerging role as a secretory organ, what is the contribution of the muscle secretome to brain health in older age?
Through which putative molecular and systems-level mechanisms might muscle-derived circulating factors influence brain health?
What are the translational challenges that need to be solved to progress the pipeline of muscle interventions and muscle secretome therapeutics to promote brain health in older adults?

### Myosteatosis: a marker of muscle quality, aging, and disease

Skeletal muscle mass, function, and quality decline with increasing age. After the age of 50, muscle mass declines in both males and in females, with reduced muscle fiber number and size, mainly due to a progressive loss of motoneurons [10]. The age-related decline in muscle quality [11], characterized by increase in myosteatosis [12], occurs earlier than the reduction in muscle mass. In addition the muscle’s architecture is modified, type II fibers decline, and muscle vasculature is reduced with aging [13].

Fat accumulation in the muscle (myosteatosis) is an important measure of muscle quality and is especially relevant in aging due to its impact on metabolism [14] and function [11]. Myosteatosis can occur in different regions with respect to the muscle fascicle, including inter-muscular (i.e., fat depots between fascicles of muscle fibers) and/or intra-muscular (i.e., fat depots within the fascicles of muscle fibers). Intermuscular adipose tissue (IMAT) is quantified by measuring the area of IMAT within a specific muscle or body region using imaging modalities such as CT, and MRI [15]. These techniques rely on differences in tissue density to distinguish fat from muscle in defined anatomical regions. Intramuscular fat depots can refer to both myocellular fat (fat within myocytes) and extramyocellular fat (fat located outside of myocytes but within muscle fascicles, between muscle fibers). Differentiating these types of intramuscular adiposity is technically challenging and requires magnetic resonance spectroscopy or muscle biopsy [16]. Imaging methods used to measure IMAT and intramuscular fat remain without strong consensus, and the similarities and differences in the development of these skeletal muscle fat depots are not well understood. Furthermore, the physiological significance of these similarities and differences remains unclear. In the literature, the term myosteatosis has been used to encompass both IMAT and intramuscular fat, and therefore, this terminology was adopted by the symposium [16–18].

Myosteatosis has local effects and can impact muscle function, including muscle contractility performance, as it relates to specific force and torque, but less known is its strong systemic effect on metabolism and link to metabolic dysfunction [19] such as insulin resistance, type 2 diabetes, hypertension, obesity [20], and systemic inflammation [16]. These are well-established risk factors for cognitive impairment and dementia in older adults [21]. Notably, myosteatosis is a strong predictor of metabolic disorders, independent of total body fat [22]. Myosteatosis also varies within populations of older adults including by sex and ethnicity. Specifically, myosteatosis tends to be higher in women than in men^1^ and may also be elevated in individuals of African and South Asian ancestry [23]. As a result, it may represent a critically important risk factor for metabolic dysfunction in these populations, particularly where measures of central adiposity do not fully account for the observed metabolic impairments [16].

Understanding the mechanisms linking myosteatosis to metabolic disorders may be critical to uncovering the impact of muscle health on brain health. It is hypothesized that the close proximity of adipose tissue to and within muscle disrupts the muscle microenvironment—either through increased local or systemic proinflammatory cytokine release or through impaired myokine secretion, which is reviewed in the next section. The inflammatory secretome associated with myosteatosis may contribute to subsequent metabolic and muscular dysfunction, particularly insulin resistance, muscle atrophy, and diminished contractility [24]. Additionally, elevated levels of myosteatosis may impair nutritive blood flow to muscle [13]. Early in this pathological process, modifications to muscle proteins and loss of coordinated regulation between contractile, mitochondrial, and sarcoplasmic reticulum proteins occur, followed by motoneuron loss [10]. This neuronal loss is inadequately compensated by reinnervation from surviving motoneurons. This cascade initiates a vicious cycle that can lead to cognitive impairment—where declining physical performance reduces physical activity, leading to less social stimulation and cognitive engagement [25].

### Interventions for myosteatosis

While numerous intervention studies have focused on increasing muscle mass and function, there is a notable lack of studies specifically targeting myosteatosis. Existing studies are limited by small sample sizes and are primarily conducted in older adults. Emerging evidence suggests that exercise is a promising strategy for reducing myosteatosis in this population. A systematic review and meta-analysis found that both aerobic exercise and combined aerobic and strength training can reduce myosteatosis, although significant heterogeneity exists in the methods used to assess it [26]. Additionally, some studies report that diet alone, or in combination with exercise, may influence myosteatosis [26]. However, additional randomized controlled trials (RCTs) are needed to evaluate the effects of various exercise modalities—such as aerobic training, high-intensity interval training (HIIT), and resistance training—as well as their combinations. These trials should also assess how differences in exercise intensity, frequency, and duration, along with dietary factors, impact myosteatosis and the associated muscle secretome.

Informed by mechanistic insights into myokine signaling, pharmaceutical interventions are also being explored. For example, drugs targeting myostatin have shown preliminary efficacy in improving muscle mass and function in phase II clinical trials, though phase III data are not yet available [27]. In mice, even short-term (4-week) treatment with an anti-myostatin antibody led to increased whole-body muscle mass, enhanced grip strength, improved insulin sensitivity, and reduced ectopic lipid accumulation [28]. While anabolic hormones are being tested in populations with myopathies, there is hesitancy to apply these treatments to older adults due to potential secondary health risks [29, 30]. In summary, RCTs that evaluate the individual and combined efficacy of exercise, pharmaceutical, diet, and lifestyle interventions on myosteatosis are urgently needed in older adults.

### Epidemiological association of muscular contractile activity and brain health

Epidemiological studies demonstrate a consistent relationship between higher physical activity levels and reduced dementia risk [13, 16, 21, 23, 25, 31]. The lack of physical activity presents the largest risk of developing late life cognitive decline and dementia among other established risk factors [32]. However, many epidemiology studies do not distinguish between different types of physical activity or exercise—a subset of physical activity that is planned, structured, and repetitive [23]. There are two main types of exercise training: aerobic exercise and resistance training, each with distinct physiological mechanisms and benefits [33]. Aerobic exercise is characterized by a sustained elevated heart rate and primarily improves cardiovascular fitness—measured by maximal oxygen uptake (VO_2_ max)—and, at the muscle level, targets type I muscle fibers to enhance carbohydrate and fat metabolism [34]. In contrast, strength and resistance training lead to fewer cardiovascular adaptations but emphasize the activation of motor units, particularly type II muscle fibers [34]. This results in improved neuromuscular synchrony and muscle fiber hypertrophy, ultimately increasing muscle strength and power. Randomized controlled trials show exercise positively impacts brain health, including neuropsychological function and brain neuroimaging indices of size and brain function [24, 25]. To date, the majority of RCTs of exercise and brain health in older adults have focused on aerobic exercise training [24]. However, the potential of resistance training for brain health is now recognized [26–28]. The benefit of resistance training on brain health can be observed among older adults who are cognitively normal as well as those with mild cognitive impairment. For example, Liu-Ambrose showed 2 ×/week resistance training improved executive function in older women with and without mild cognitive impairment [4, 6]. Moreover, 2 ×/week resistance training reduced the progression of cerebral small vessel disease [9]—a condition that contributes to 45% of dementia cases [29]. Recent reports show that resistance training significantly improves memory, preserves white matter integrity, and maintains hippocampal grey matter in older adults with mild cognitive impairment, compared to a unimpaired control group, following a 24-week intervention [35]. The effects of different exercise training (i.e., aerobic training and resistance training) on the muscle secretome remain to be elucidated, particularly among older adults. Evidence to date from studies of acute exercise suggests both types of exercise training induce changes in myokine levels in healthy adults [30]. Overall, there is a general dearth of research examining the muscle secretome response to acute and chronic resistance exercise in older adults.

### Mechanisms of muscle-brain crosstalk: circadian rhythms, muscle clocks, myokines, and extracellular vesicles

#### Circadian rhythms and muscle clocks: a potential systems biology link supporting muscle to brain bidirectional communication

Circadian rhythms have evolved to synchronize physiology and behavior with the 24-h rotation of the Earth. These rhythms are governed by an intrinsic molecular clock, present in nearly every mammalian cell, which is aligned to the external light–dark cycle. Population studies have shown that irregular and insufficient sleep are strong, independent risk factors for mild cognitive impairment (MCI) and incident dementia [36]. Irregular sleep and activity patterns have been linked to disrupted circadian rhythm integrity, which is thought to be a significant contributor to declining brain health, as measured by cognitive performance, cerebrospinal fluid (CSF) biomarkers, and neuroimaging findings [37–39]. Given that physical activity is a key factor influencing circadian rhythm integrity—and that skeletal muscle plays a central role in generating physical activity—it is plausible that muscle itself may contribute to the regulation of the circadian behaviors. Moreover, the growing body of evidence linking circadian rhythms to both brain health and physical activity supports a systems biology rationale for investigating the interactions between the musculoskeletal system and the central nervous system. Specifically, this line of inquiry may help determine whether circadian clock mechanisms serve as a biological link connecting musculoskeletal health to central nervous system function.

Some of the earliest work to identify muscle-specific circadian clock output genes collected muscle tissue every 4 h over a 24-h period in order to identify genes exhibiting circadian expression patterns [40]. Specifically, this resulted in approximately 215 mRNAs expressed in a circadian manner in skeletal muscle, including core clock genes such as *Bmal1*, *Per2*, and *Cry2* [40]. Over time, mounting evidence has shown that the genetic regulators of the core circadian clock mechanism in mouse and human skeletal muscle, as well as downstream clock-controlled outputs, are highly conserved across species [41]. Comparative studies of genes expressed in a circadian pattern have identified key muscle-specific transcription factors such as *MyoD1*, as well as genes essential for glucose metabolism, including hexokinase and Glut4 [41]. In relation to muscle–brain communication, it has also been observed that *Fndc5*, the precursor to the myokine irisin, is a clock output gene in skeletal muscle as are other genes that contribute to extracellular vesicle formation and transport [42]. These observations support the importance of the muscle clock beyond muscle strength and metabolism, and they highlight potential molecular targets that modify muscle to brain communication [42–44].

A novel investigation advanced the understanding of skeletal muscle’s potential role in brain health by demonstrating that skeletal muscle is a key contributor to features of non-rapid eye movement (NREM) sleep in mice [45]. Specifically, restoration of *Bmal1* expression in skeletal muscle tissue alone was sufficient to rescue both the total amount of NREM sleep and the slow-wave activity recovery response following sleep deprivation in *Bmal1* knockout mice [45]. These findings underscore the critical role of skeletal muscle—and in particular, the molecular clock gene *Bmal1* within muscle—in maintaining both sleep quantity and quality. Although the downstream molecular mechanisms linking skeletal muscle to the regulation of NREM sleep remain unclear, this work highlights a novel communication pathway between the musculoskeletal system and the central nervous system, particularly the brain.

Given the critical role of skeletal *Bmal1* in maintaining circadian integrity and regulating sleep, several research groups have extended investigations to examine the impact of restoring *Bmal1* specifically in skeletal muscle on additional outcomes in *Bmal1* knockout mice. The mice lacking Bmal1 in all cells of the body have been proposed as a model of advanced aging with diminished cage activity and a significantly reduced lifespan [46]. Restoration of skeletal muscle *Bmal1* has been associated with extending the *Bmal1* KO mouse lifespan, improved systemic glucose homeostasis, and reduced levels of systemic inflammation [42, 47, 48]. Additionally, mice with restored muscle *Bmal1* exhibited increased voluntary wheel-running activity, suggesting that improved muscle health can influence the brain to modify activity behavior [45]. These changes in muscle function and behavior were accompanied by improved markers of tissue and systemic health, including enhanced metabolic profiles and reduced inflammation in both the lungs and white adipose tissue [47]. Although the brain was not directly studied in these experiments, the findings highlight the capacity of healthy muscle to communicate with other biological systems to support both physical activity and overall health.

In summary, studies of the circadian clock mechanism in skeletal muscle have revealed new insights into how muscle-intrinsic circadian regulation contributes not only to muscle health, but also to broader systemic and brain health.

#### Molecular messengers of muscle-brain crosstalk: the role of myokines

Skeletal muscle is a highly active endocrine organ with a complex secretome that includes nucleic acids, fatty acids, and—most notably—bioactive peptides and proteins collectively known as myokines [5]. These may be secreted directly or encapsulated within EVs [49]. To date, over a thousand putative myokines have been identified; however, only a small subset has been consistently examined in human population-based studies, with many others remaining promising but largely investigated in animal models or in vitro [50]. The majority of identified myokines have also been previously classified as cytokines. These molecules are not secreted exclusively by myocytes but are also produced by adipocytes located within muscle and other fat depots—leading to the term adipomyokines in some contexts [51]. Furthermore, there is considerable overlap between the muscle secretome and those of other organs, particularly the liver [5].

Myokines are thought to play a critical role in maintaining whole-body metabolic homeostasis through autocrine, paracrine, and endocrine signaling, influencing nearly every organ system—including the brain.5 Among the diverse mechanisms underlying muscle–brain interactions are metabolic regulation, immune modulation, cerebrovascular health, and direct neurotrophic effects. Myokines most frequently associated with both metabolic and brain health include monocyte chemoattractant protein-1 (MCP-1), interleukin-6 (IL-6), Cathepsin B, myostatin, fibronectin type III domain-containing protein 5 (FNDC5)/irisin, and brain-derived neurotrophic factor (BDNF) [45–50]. Since the discovery that IL-6—a known cytokine—is also produced and released by skeletal muscle, interest has grown in the crosstalk between muscle and brain as a key pathway for understanding how exercise promotes systemic health [31].

The following sections will review myokines linked to brain health, beginning with evidence from population-based studies, followed by their relationship to biomarkers and mechanisms associated with cognitive decline and AD.

#### Myokines and cognition in human population studies

Higher plasma levels of MCP-1 were associated with the following: lower scores and faster decline in episodic memory in cognitively normal adults [52, 53], faster conversion from MCI to dementia with potential underlying AD [54], AD, dementia with potential underlying AD, or MCI status compared to normal cognition [55–58], and faster cognitive decline in patients with MCI or dementia with potential underlying AD [59]. Patterns of associations were similar for studies using other measures of MCP-1 (e.g., gene polymorphism [60], and CSF levels [61, 62]). As a myokine, IL-6 has differential effects depending on the chronicity of its elevation. Acute increases in IL-6 following muscle contraction promote glucose uptake, lipolysis, and activation of anti-inflammatory pathways [5]. In contrast, chronically elevated IL-6 levels, particularly when accompanied by larger levels of intraindividual variability, are associated with cognitive decline [63–66]. Higher circulating levels of Cathepsin B, as well as greater increases in serum Cathepsin B concentrations, exhibit potential associations with improvements in cognitive function in response to exercise. However, existing studies have not demonstrated consistent relationships between increased Cathepsin B levels and improvements in cognition, and they show considerable heterogeneity in both the type and duration of exercise regimens as well as in the cognitive assessments used [67, 68]. Recent reviews have highlighted the need for greater consensus in study designs and have emphasized the importance of larger, longitudinal studies examining Cathepsin B and its association with cognition at the population level in older adults [69]. Myostatin has been associated with a neuroprotective phenotype with sustained cognitive function, both cross-sectionally and longitudinally, in a large epidemiological study of older adults without dementia [70]. In a separate study, myostatin has showed no relationship with cognitive function but was positively associated with incident dementia with potential underlying AD [71]. It is difficult to interpret these conflicting results given the limited number of existing studies and the possibility that an increase in myostatin may represent an adaptive or compensatory response in older adults with incident dementia. These findings underscore the need for further research at the population level.

Higher plasma irisin was associated with better global cognition and episodic memory among middle aged adults with familial history of AD [72], and with normal cognition compared to dementia with potential underlying AD [73, 74]. In apparent contrast with these findings, a study of patients aged 45–75 with uncontrolled type II diabetes (T2D) found plasma irisin was higher in MCI compared to healthy unimpaired controls and was inversely correlated with cognition [75]. As mentioned previously, it has been suggested that myokine levels increase as adaptive responses to dysfunction in metabolism; for example, irisin may increase in patients with type II diabetes mellitus as an adaptive/compensatory response, but its neuroprotective effects may not be sufficient to combat the neurodegenerative effects of pro-inflammatory adipokines. Most prior studies tested these cytokines in isolation, which thus could not assess how they interact with each other. Studies in humans were mostly conducted in US and Asia and did not include persons of African ancestry. Future research proposals should address these limitations. Irisin is a particularly interesting myokine because it has been shown to have neuroprotective effects [76], in particular in the hippocampus, by increasing BDNF levels [77].

While most BDNF research has focused on its endogenous effects on neural plasticity in preclinical models, BDNF is also known to be expressed in muscle in response to exercise [78] and is present in measurable amounts in plasma and serum [79]. This raises the possibility that BDNF may mediate a direct muscle-to-brain signaling pathway. Studies measuring serum BDNF levels have shown that lower circulating BDNF is associated with dementia with potential underlying AD compared to cognitively healthy, unimpaired older adults [80, 81]. However, serum BDNF levels do not appear to reliably distinguish between individuals with MCI and healthy unimpaired controls, or between those with dementia with potential underlying AD and MCI. BDNF levels vary greatly from individual to individual and are influenced by sex, physical activity, diet, sleep, stress, and other environmental factors. A limitation of current studies is that, while some have measured cognition longitudinally, many have not measured within-subject BDNF levels over time. Tracking individual changes in BDNF could offer additional insight. Another limitation involves the plausibility of whether BDNF, a serum protein, can cross the blood–brain barrier; BDNF present in the bloodstream arises from endothelial cells and peripheral tissues and is stored in platelets [79]. These issues raise the possibility that serum or plasma BDNF may instead serve as a surrogate marker of physical function or muscle health. An alternative mechanism is that muscle-derived EVs may encapsulate BDNF and other molecular cargo, potentially allowing them to cross the blood–brain barrier and interact directly with the brain.

#### Myokines, biological biomarkers of dementia, and mechanisms of muscle-brain interactions in human studies

Information on myokines, AD pathology, and muscle-brain interactions is supported by animal studies, but is only recently emerging in humans. Plasma MCP-1 was positively associated with CSF levels of phosphorylated and total tau in asymptomatic older adults [62]. Exposure of human cell cultures to MCP-1 resulted in increased Tau phosphorylation [82]. Patterns of associations were overall similar for CSF levels of irisin and MCP-1 predicting CSF levels of AD biomarkers [58, 61, 62, 77]. MCP-1 plays an important role in regulating vascular permeability, and it is hypothesized that elevated circulating levels of MCP-1 may alter the selectivity of the blood–brain barrier. Supporting this, endothelial cells from individuals with AD show increased levels of inflammatory factors, including MCP-1, compared to those from individuals without AD. Notably, high concentrations of MCP-1 have been observed in preclinical models within the brain microvasculature of individuals with AD, but not in those without AD [83]. Increased permeability of the blood–brain barrier may expose the brain to factors that contribute to cognitive decline, including inflammatory mediators and circulating amyloid.

Chronically elevated serum levels of IL-6, similar to MCP-1, have been associated with an inflammation-mediating role in AD. For example, both IL-6 and MCP-1 have shown strong correlations between serum and CSF levels in individuals with probable AD [84]. This supports the hypothesis that increased blood–brain barrier permeability may allow serum inflammatory mediators to access the CSF and, consequently, the brain—thereby promoting inflammatory pathways, a recognized hallmark of AD [85]. Another possibility involving IL-6 is that exercise is known to lead to large increases in IL-6, and in preclinical mouse models, elevated IL-6 has been shown to induce reduced appetite [86]. This may have important implications given the strong link between metabolic syndrome and cognitive decline. These findings suggest that both chronic elevations and activity-induced fluctuations in serum IL-6 levels could contribute to distinct cognitive phenotypes.

Cathepsin B is a myokine known to be released directly from skeletal muscle in response to both aerobic and strength-based exercise regimens in human populations. In rodent models, Cathepsin B has been shown to cross the blood–brain barrier [87, 88]. It influences neural substrates vulnerable in dementia, particularly the hippocampus and prefrontal cortex, and promotes neural plasticity by enhancing neurogenesis and synaptic connectivity. Cathepsin B also contributes to the degradation of amyloid-beta, directly interacting with AD pathological mechanisms. The pathways through which Cathepsin B enhances neurogenesis and synaptic function are multifaceted and include the proliferation and differentiation of neural progenitor cells, axonal growth, reduction of neuroinflammation, and induction of local BDNF expression [69, 87]. The effect of Cathepsin B on amyloid-beta accumulation is likely mediated through increased lysosomal activity that supports cellular recycling [69, 89].

In human studies, higher circulating levels of myostatin have been associated with higher plasma Aβ42/40 ratios, which are indicative of lower amyloid burden [90]. Additionally, elevated serum myostatin levels have been linked to more favorable neuroimaging profiles and reduced amyloid deposition as measured by PET scans [70]. Although research in this area is still limited, these findings suggest a potential neuroprotective role for myostatin. Mechanistically, impaired autophagy and disrupted protein degradation are thought to contribute to the accumulation of amyloid plaques and neurofibrillary tangles. Therefore, it is biologically plausible that myostatin, by decreasing protein synthesis and/or enhancing autophagy pathways through mechanisms such as protein ubiquitination, may be associated with reduced amyloid accumulation.

Plasma levels of irisin in middle-aged adults with familial AD were inversely associated with serum amyloid Aβ42, although this association only approached statistical significance—likely due to the small sample size (*N* = 64) and relatively young average age (55 years) of participants [91]. In CSF, irisin levels correlated with several biomarkers of AD pathology, including a positive correlation with CSF Aβ42 (indicative of lower central nervous system amyloid burden), a positive association with BDNF, and a negative trend with total tau (t-tau) [77, 92]. Preclinical work using 3D brain organoid models suggests that irisin may reduce amyloid burden through activation of the Aβ-degrading enzyme neprilysin [93]. As a myokine, irisin has garnered considerable attention due to its ability to influence multiple brain cell types—neurons, astrocytes, and microglia—and activate a range of neuroprotective molecular pathways, including those regulating amyloid pathogenesis, neuroinflammation, mitochondrial function, and BDNF production [94]. Particularly relevant to the muscle-brain axis, peripheral irisin expression has been shown to drive BDNF production in the brain in response to exercise in mouse models [64]. Therefore, elucidating the mechanisms by which FNDC5/irisin exerts its effects is critical for developing therapeutic strategies to mitigate cognitive dysfunction—especially for individuals who are frail, injured, or otherwise unable to engage in physical activity.

BDNF is a key molecule that promotes neurogenesis, dendritic and synaptic health, neuronal survival, plasticity, and excitability, all of which are disrupted in neurological and cognitive disorders, including AD. While BDNF is released by skeletal muscle, it is thought to act primarily through autocrine mechanisms. Nonetheless, BDNF remains central to investigations of muscle-brain interactions, as physical activity—and the associated circulation of myokines and EV cargo—is believed to influence BDNF production within the brain. Consequently, BDNF is increasingly viewed as a biomarker for cognitive decline and resilience. Although muscle may not contribute substantially to serum BDNF levels, significant input comes from platelets, endothelial cells, and possibly the brain itself via blood–brain barrier crossing [95]. Importantly, BDNF not only promotes direct neuroprotection but also has bidirectional relationships with AD-related mechanisms involving tauopathy and amyloid pathology, reinforcing its relevance in studies of muscle-brain mechanisms [96, 97].

##### Extracellular vesicles as carriers of molecular communication from muscle to brain

Extracellular vesicles (EVs) are present in fluids throughout the body, including the blood, urine, lymphatic fluids, and saliva. Notably, these EVs serve as critical carriers of information in the form of nucleic acids, proteins, and lipids, for example. As they circulate through the fluid, EVs are eventually taken up by a recipient cell, where they can dictate physiological cascades and/or pathological processes. Recent work demonstrated that young serum exerts beneficial effects on both skeletal muscle regenerative potential and cognitive function in aged mice, but that the effects were significantly blunted when the serum was depleted of circulating EVs. [98, 99] Further investigation demonstrated that young EVs enhanced the mitochondrial function of aged muscle progenitor cells, a major cell population that participates in the skeletal muscle regenerative cascade [98]. These data suggest that mitochondria may be a target of circulating EVs, and that age-related declines in the cargoes of these EVs may contribute to the loss of tissue health over time. This raises the intriguing possibility that EVs may serve as mediators of the muscle brain-axis and that interventions such as exercise or muscle contractile activity may serve as a promising means to reverse the effect of time on the information contained within these nanoparticles. Indeed, we recently demonstrated that 2 weeks of neuromuscular electrical stimulation performed in aged animals significantly altered EV cargoes and enhanced their ability to support skeletal muscle regeneration [100]. Future studies are needed to identify the molecular mediators of these effects.

EVs offer a promising avenue to study the full secretome of skeletal muscle, particularly in relation to their potential targeting of the brain. The encapsulated cargo within EVs protects nucleic acids including small RNAs with signaling capacity, enables identification of the tissue of origin, and stabilizes bioactive lipids that may otherwise degrade in free circulation [101]. While these techniques are not yet fully developed for muscle-derived EVs, they are rapidly emerging in the context of brain-derived EVs, with growing number of reports detailing methods for isolating and identifying brain-derived EVs from the circulation [49, 102–104]. These approaches could be applied to identify muscle-derived EVs and potentially those specifically targeted to the brain. Moreover, the detection of brain-derived EVs in systemic circulation supports the biological plausibility that EVs can cross the blood–brain barrier and protect their molecular cargo [102]. Future research integrating EVs from muscle and brain, and translating brain EV isolation techniques to muscle, holds substantial promise for advancing our understanding of muscle-brain interactions.

## Discussion/conclusions

The symposium, *The Role of Muscle-Brain Crosstalk to Promote Healthy Aging*, aimed to elucidate current evidence, limitations, and future directions for understanding muscle’s role in brain health. Discussions centered on four key topics, each providing take-home messages, identifying limitations, and cross-cutting themes. A primary and recurring recommendation across sessions was the need for further exploration of the emerging relationship between muscle quality and brain health. In particular, there was strong emphasis on the need for investigating muscle’s secretome of circulating factors—such as myokines and EVs—as mechanistic links in muscle-brain crosstalk.

### Myosteatosis: a marker of muscle quality, aging, and disease

The first topic of the symposia, *Muscle Quality in Aging and Disease*, established that while past assessments of muscle have primarily focused on muscle mass (e.g. muscle area) and strength, muscle quality is one of the earliest changes in aging and strongly impacts muscle strength, the risk of metabolic disorders (e.g., insulin resistance), and systemic inflammation [11, 12, 16, 17]. These factors, in turn, have well-established causal relationships with cognition and dementia. This supports the biologic plausibility that muscle quality, measured by myosteatosis, may play a significant role in brain health.

A recommendation from this session was the need for further investigation into myosteatosis as a predictor for cognition and brain health in aging populations. Additionally, there is a critical need to develop targeted therapies and interventions that address myosteatosis in conjunction with muscle mass and function. However, progress in effectively targeting myosteatosis is hindered by three major limitations: (1) the lack of consensus on standardized morphological measurement methodologies (e.g., ultrasound, computed tomography, MRI) for myosteatosis, (2) the relevance of intermuscular and intramuscular skeletal fat depots, and (3) the absence of muscle-derived biomarkers associated with myosteatosis.

A cross-cutting theme that emerged early in the symposia was the importance of understanding the muscle pathophysiology underlying myosteatosis, particularly the role of myokines. In this context, secreted mediators such as myokines and the cargo of EVs have been proposed as potential molecular biomarkers. This is especially relevant given that current assessments of myosteatosis primarily rely on imaging-based morphometric analyses.

### Epidemiological association of muscle contractility and brain health

The second topic, *Epidemiological Association of Muscle Contractility and Brain Health*, highlighted the critical role of muscle function in enabling physical activity. However, the direct link between muscle health characteristics, muscle biology, and brain health remains underdeveloped. A key emphasis was that most exercise interventions focus on endurance and sustained aerobic exercise, which primarily targets substrate metabolism but may have limited direct effects on myosteatosis. In contrast, resistance training, which influences changes to muscle fiber density, structure, and the muscle’s secretome, have been less studied in the context of brain health [17, 105].

Recommendations to advance muscle contractility’s association to brain health is summarized by the need for clinical trials directly comparing resistance training and aerobic exercise to assess their differential impacts on brain health. These trials should simultaneously evaluate both the acute and chronic responses of myokines and EVs and their relationship to cognitive and brain health outcomes. Future research should also adopt standardized definitions of brain health, such as those proposed by the Alzheimer’s Association and NIA, which integrate cognitive impairment and biomarkers to classify stages including unimpaired AD, mild cognitive impairment, and the spectrum of dementia with potential AD and AD [106]. Additionally, resistance training protocols should be refined to distinguish between power, strength, and hypertrophy-focused interventions using established variables in exercise prescription, as each may have distinct effects on myosteatosis and neuroprotection [24].

Building on the first symposia’s recommendations, the discussion of Muscle Contractility’s association with brain health reinforced the themes of understanding the mechanistic role of muscle quality—specifically myosteatosis—in brain health. Further, the integration of myokine and EV biology into exercise research is essential to uncover molecular pathways linking muscle contractility to neuroprotection. Given the reliance on imaging-based assessments for myosteatosis, identifying myokine and extracellular vesicle biomarkers could provide more accessible and scalable methods to track muscle-related cognitive benefits.

### Mechanisms of muscle-brain crosstalk: circadian rhythms, muscle clocks, myokines, and extracellular vesicles

#### Circadian rhythms and muscle clocks

The third topic, Circadian Rhythms and Muscle Clocks, explored the biologic plausibility of skeletal muscle’s role in brain health through a systems biology perspective. A key insight emerging from this discussion is that skeletal muscle circadian clocks play a crucial role in maintaining overall circadian rhythm integrity, which is increasingly recognized as a factor in cognitive health [42, 45].Disruptions in circadian rhythms due to aging, declining physical activity, or night shift work have been linked to cognitive decline and dementia [37, 37, 38]. Skeletal muscle health, particularly the regulation of its molecular pathways (e.g., BMAL1), is fundamental to preserving circadian function. While the relationship between myosteatosis and skeletal muscle clocks remains largely unexplored, this perspective further reinforces the role of skeletal muscle in neuroprotection beyond its cardiovascular, metabolic, and inflammatory effects.

Further research is needed to determine how skeletal muscle contributes to system-wide circadian regulation and whether the molecular mediators involved in circadian signaling differ from those influencing cardiovascular, metabolic, and neurologic outcomes. Additionally, studies should evaluate how myosteatosis interacts with skeletal muscle clocks to impact brain function. A major limitation in advancing this field is the lack of research on specific biomarkers linking skeletal muscle circadian function to brain health.

Building on previous symposia discussions, this topic reinforces the critical role of skeletal muscle beyond traditional metabolic and functional perspectives, integrating circadian biology into the broader discussion of muscle’s neuroprotective potential. Additionally, it expands the scope of potential interventions beyond exercise alone to include diet, sleep regulation, and the timing of physical activity as strategies to optimize muscle quality and its impact on brain health. Given that myosteatosis assessments currently rely on imaging, identifying circadian-related biomarkers in muscle-derived factors such as myokines and EVs could offer novel and accessible tools for tracking muscle’s influence on neuroprotection.

### Molecular mechanisms of muscle-brain interactions

The fourth topic, Molecular Mechanisms of Muscle-Brain Interactions, focused on two key objectives: (1) strengthening the causal relationship between muscle and brain health and (2) identifying molecular targets for translational applications, including exercise and pharmacologic interventions. The primary mechanisms underlying muscle-brain interactions include effects on metabolism, immune function, cerebrovascular health, and direct neural pathways. Skeletal muscle secretes myokines and EVs containing cargo such as nucleic acids and fatty acids, with neuroactive myokines—such as MCP-1, IL-6, Cathepsin B, myostatin, irisin, and BDNF—receiving significant attention [5]. These molecules have been implicated in brain health through both preclinical studies and associations in older adult populations. EVs are of particular interest due to their biological advantages: they protect molecular cargo with a plasma membrane, contain proteins that may aid in targeting, and have demonstrated the ability to cross the blood–brain barrier in preclinical models. This presents an exciting avenue for understanding and potentially modulating muscle-derived influences on neuroplasticity and brain function.

One major challenge in studying myokines and EVs is determining their tissue or cellular origin, as many of these molecules are not muscle-specific. For example, interventions such as resistance training may elevate serum levels of irisin or BDNF, but it remains difficult to distinguish whether these effects result from muscle signaling or secondary activation of the brain’s endogenous BDNF production. Additionally, there is ongoing debate about whether irisin or BDNF can cross the blood–brain barrier. Innovative methodologies are needed to enhance the interpretation of interventional studies investigating the effects of muscle-targeted interventions on myokines and EVs, neuroplasticity, and cognition.

EVs present a promising intervention strategy, supported by ongoing advances in identifying their surface proteins and profiling their cargo. Clinically, EVs are of particular interest because they can cross the blood–brain barrier and be engineered with surface proteins to target specific payloads [107]. As a result, they are being actively explored as therapeutic candidates in brain disorders such as glioblastoma (GBM) and stroke recovery [108]. This therapeutic potential could also be extended to neurodegenerative conditions, including AD.

To accelerate progress, the development of multidisciplinary research teams is crucial. Given that EVs appear to participate in bidirectional communication between the brain and periphery—with the discovery of brain-derived EVs—there is an emerging potential for EVs to serve not only as therapeutic vectors but also as molecular readouts, akin to a “liquid brain biopsy” [102]. Collaborative teams integrating expertise in skeletal muscle physiology, myosteatosis assessment, EV biology, aging research, and multimodal brain assessments would significantly advance the field.

This discussion expands on prior symposia topics by reinforcing the biological plausibility of skeletal muscle’s role in brain health, expanding from its metabolic, cardiovascular, and circadian influences to molecular-level interactions. Additionally, it highlights EVs as a novel biomarker and therapeutic strategy, aligning with the broader need to refine molecular readouts of muscle function beyond imaging-based assessments of myosteatosis. Further, the potential for bidirectional brain-muscle communication through EVs underscores the need for integrative research approaches that connect muscle biology with neurodegenerative and cognitive health frameworks.

### Next steps for research: mechanisms, interventions, and the necessity of multidisciplinary collaboration

**Fig. 2:**
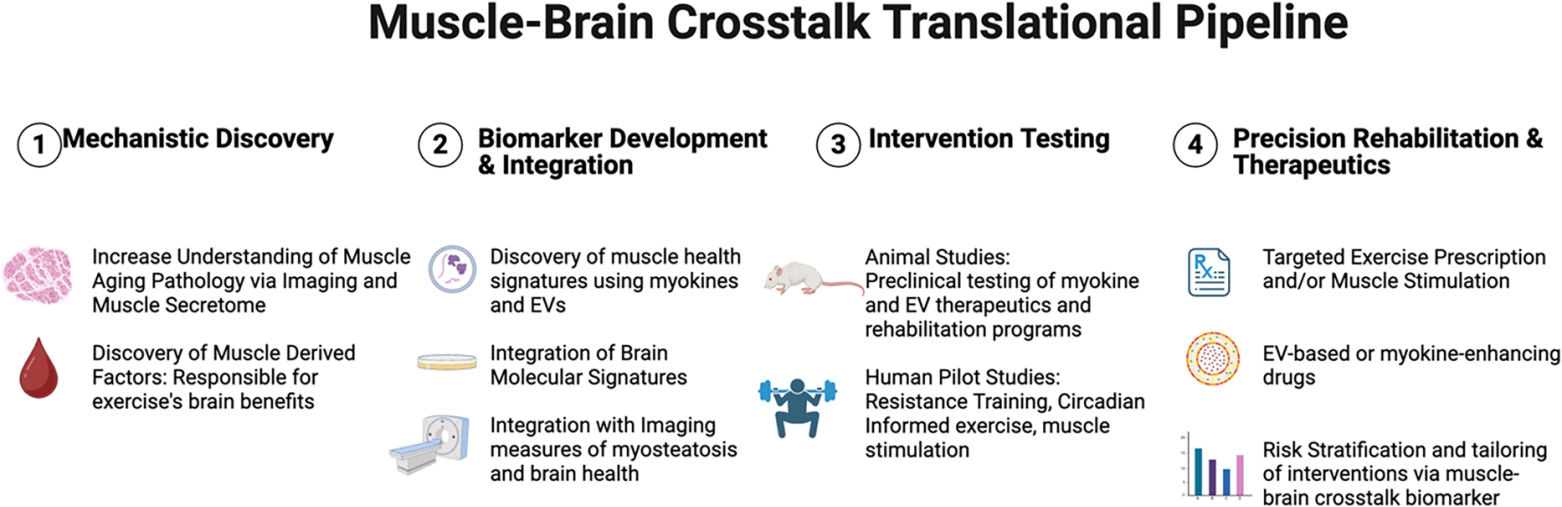
Translational pipeline for muscle–brain crosstalk research. This model maps the proposed research trajectory—from mechanistic discovery to biomarker integration, intervention testing, and precision therapeutics—to highlight potential clinical impact

Figure 2 illustrates a proposed translational pipeline for muscle–brain crosstalk research, from mechanistic discovery to precision therapeutics. Specifically, future research must prioritize the precise characterization and measurement of myosteatosis, myokines, and EVs as well as their cargo to establish a clearer understanding of the muscle secretome and its role in brain health. A critical step toward this goal is the development of consensus methodologies for assessing myosteatosis, particularly differentiating between intermuscular and intramuscular fat depots and defining their specific relevance to brain health. Standardizing imaging techniques (e.g., CT, ultrasound, MRI) alongside molecular profiling (e.g., myokine and EV cargo signatures) will be essential to capturing muscle health at both macroscopic and molecular levels. Advancements in proteomics and transcriptomics should be leveraged to better identify the muscle-derived secretome with greater specificity and differentiate muscle-derived factors from those originating in other tissues.

Understanding the putative mechanisms linking muscle health to cognitive function requires an integrative, systems-level approach that considers cardiovascular, metabolic (e.g., insulin sensitivity), inflammatory, neurotrophic, and blood–brain barrier permeability mechanisms. Special attention should be given to the circadian regulation of muscle function, as skeletal muscle clocks have emerged as key regulators of systemic circadian rhythms, metabolism, and brain health. Future research should explore how disruptions in muscle circadian rhythms contribute to cognitive decline and whether interventions targeting circadian alignment (e.g., optimized exercise timing, sleep regulation, or meal timing) can enhance both muscle health and neuroprotection.

To establish causal relationships, longitudinal and interventional studies are needed to assess the direct effects of myokines and EVs on brain health. While epidemiological data strongly support the link between physical activity and brain health, definitive evidence that muscle-derived factors mediate these effects is still lacking. Clinical and preclinical studies should investigate how interventions—such as exercise prescription, drugs, dietary modifications, and sleep optimization—modulate muscle clocks and influence brain aging. The use of animal models, including genetically engineered mouse models, can provide insight into how EVs are secreted, transported, and act on distant tissues. Additionally, innovative tracking techniques for EV circulation and brain uptake will be necessary to determine their mechanistic contributions to neuroprotection.

To harness the translational potential of muscle brain crosstalk, future studies should leverage randomized controlled trials that investigate exercise interventions, particularly resistance training, and assess changes in circulating myokines, EV content, and brain health outcomes. A particularly promising avenue is the engineering of muscle-derived EVs loaded with neuroprotective cargo—such as BDNF, irisin, and neurotrophic small RNAs—for potential therapeutic applications in neurodegenerative diseases. The ability of muscle-derived EVs to cross the blood–brain barrier suggests their potential as both biomarkers (“liquid brain biopsy”) and delivery vehicles for targeted therapeutics. To accelerate clinical translation, collaboration is needed among experts in muscle physiology, myosteatosis, aging, omics, and multimodal assessments of brain health. This includes integrating neuropsychological evaluation, brain biomarker analysis, and neuroimaging to establish robust associations and identify causative mechanisms. Such interdisciplinary efforts will help lay the groundwork for developing muscle–brain crosstalk therapies for neurodegenerative conditions. Additionally, these findings could inform public health policies by reinforcing the role of muscle health and physical activity in cognitive aging and dementia risk modulation, guiding the development of exercise and rehabilitation strategies tailored to enhance brain health in aging adults. To support multidisciplinary collaboration, funding agencies should structure funding notices to promote research that advances understanding of muscle–brain crosstalk at the molecular level, while simultaneously supporting clinical investigations—including exercise, lifestyle, and pharmaceutical interventions—to improve muscle quality, brain health, and inform clinical trials.

The highlighted and aforementioned next steps for research are summarized in Fig. 2, which outlines a proposed translational pipeline for muscle–brain crosstalk—from mechanistic discovery to biomarker development, intervention testing, and precision therapeutics.

### Symposiums impact

The symposium, *The Role of Muscle–Brain Crosstalk to Promote Healthy Aging,* elucidated the current state of the field and examined the underlying mechanisms linking muscle–brain crosstalk to the well-established relationship between physical activity and brain health. Particular emphasis was placed on the limitations of the existing research base and the opportunities for future studies to strengthen the evidence supporting this emerging area. Given the growing associations between muscle quality—specifically myosteatosis—and cognitive function across diverse cohorts of older adults, the lasting impact of the symposium is its call for the formation of multidisciplinary teams to rigorously investigate the molecular mediators of muscle–brain crosstalk, with special focus on myokines and EVs.

With the advent of multi-omic analytic approaches, this next step is increasingly feasible and essential for uncovering the mechanisms and molecular pathways involved in neuroprotection and cognitive risk. Identifying a neuroprotective myokine and EV signature could enable the development of novel therapeutic studies—for example, engineering EVs with targeted bioactive cargo designed to reach the brain. Ultimately, the rehabilitation and therapeutic strategies informed by this research could transform how we address aging muscle and brain in older adults.

## Supplementary Information

Below is the link to the electronic supplementary material.

Supplementary file1 (DOCX 14 KB)

## Data Availability

Data is available from reasonable request from the Health-ABC study.
