# Supplementary material for: Muscle-brain crosstalk as a driver of brain health in aging
Source: GeroScience. 2025 Aug 15;48(3):4873–91. doi: 10.1007/s11357-025-01833-0 (PMC13356145; doi:10.1007/s11357-025-01833-0)
Supplement: Supplementary file 1 — Supplementary file1 (DOCX 14 KB) [file 11357_2025_1833_MOESM1_ESM.docx]

**Supplementary Table 1: Presenters and Associated Symposium Sections**

| **Presenter** | **Section** | **Topic** |
| --- | --- | --- |
| Iva Miljkovic | Section 1 | Myosteatosis: A marker of muscle quality, aging, and disease |
| Teresa Liu-Ambrose | Section 2 | Epidemiological Association of Muscular Contractile Activity and Brain Health |
| Karyn Esser | Section 3.1 | Circadian rhythms & muscle clocks: a potential systems biology link supporting muscle–brain bidirectional communication |
| Caterina Rosano | Sections 3.2.1 & 3.2.2 | Molecular messengers of muscle-brain crosstalk: the role of myokines; Myokines and cognition in human population studies |
| Margaret Fahnestock | Section 3.2.3 | Myokines, biological biomarkers of dementia, and mechanisms of muscle-brain interactions in human studies |
| Fabrisia Ambrosio | Section 3.3 | Extracellular vesicles as carriers of molecular communication from muscle to brain |
